# An alternative index to the global competitiveness index

**DOI:** 10.1371/journal.pone.0265045

**Published:** 2022-03-21

**Authors:** María-Dolores Benítez-Márquez, Eva M. Sánchez-Teba, Isabel Coronado-Maldonado

**Affiliations:** 1 Department of Applied Economics (Statistics and Econometrics), Faculty of Economics and Business, University of Malaga, Malaga, Spain; 2 Department of Business Management, Faculty of Economics and Business, University of Malaga, Malaga, Spain; Chung Shan Medical University, TAIWAN

## Abstract

This paper reviews the methodology used by the World Economic Forum (WEF) to create the Global Competitiveness Index (WEF-GCI). We propose an alternative competitiveness index that only includes the objective data (hard data) from the WEF-GCI and is created by applying a multivariate statistical procedure (Exploratory Factor Analysis) that allows us to determine the weights from the implicit data structure. The rankings obtained from this index have a high degree of association with those provided by the WEF. The main benefit of this index over the WEF index is that it does not include valuations from opinion surveys given to business executives and/or entrepreneurs of the countries included in the index (soft data). Consequently, the rankings from this alternative index are not affected by political biases or individual interests as it is elaborated only including officially published objective data.

## 1. Introduction

The competitiveness of a country or nation, which is the subject of this paper, is a controversial concept that has been under discussion for decades. The scientific literature on the subject offers various definitions that emphasize some of the multiple factors that may be related. Sustained economic growth, political stability, financial and banking infrastructure, the strength of exports, natural resources, the soundness of the government, and the education system, among others, affect a country’s competitiveness [1].

In terms of countries, one of the leading, most quoted definitions of competitiveness can be found in the Report of the President’s Commission on Competitiveness [2, p. 6], a definition which matches with the Organization for Economic Co-operation and Development (OECD). It is described as ‘‘the degree to which a country can, under free and fair market conditions, produce goods and services which meet the test of international markets, while simultaneously maintaining and expanding the real income of its people over the longer term” [3, p. 237]. This definition refers to the competitiveness of a nation as a whole, in order to be framed within a context of macroeconomic policy, and states that the ultimate goal of a nation’s competitiveness is to improve the standard of living and real income of its citizens, which can be achieved by offering goods and services at internationally competitive [4, 5].

Other definitions of competitiveness include, although not limited to, those proposed by several organizations or scholars [3, 6–9], which also include the country’s capacity to create a favorable environment for improving the wealth and welfare of its citizens.

According to Porter [8, p. 167], “the only meaningful concept of competitiveness at the national level is national productivity.” He also points out that the main goal of a nation is to establish the necessary conditions to improve the standard of living of its citizens, that the ability to do so depends on productivity, and that it is related to how a nation uses its employment and capital. Fagerberg [6, p. 355] defines competitiveness as “the ability of a country to realize central economic policy goals, especially growth in income and employment, without running into balance-of-payments difficulties.” The same author later stated that an agreed definition of international competitiveness could be “the ability of a country to secure a higher standard of living for its citizens than comparable economies for the present and the future” [7, p. 48]. Furthermore, in regard to the idea of reaching a consensus, Aiginger [9, p.174] proposes defining competitiveness as a country’s “ability to create welfare.” He also suggests that a comprehensive assessment of international competitiveness should take into account both the result, which measures the country’s performance and is closely related to welfare, and the process (which investigates factors affecting the generation of the result).

Every year, organizations such as the World Economic Forum (WEF), the Institute for Management Development (IMD) and the Centre for International Competitiveness from the University of Wales (UK) (CICUW) publish lists in which countries are classified in order of their competitiveness; i.e. competitiveness rankings. These rankings are used by policymakers and stakeholders to judge the relative success of their country in meeting the competitiveness criteria used in the corresponding competitiveness index.

These three indices share the philosophy that a country’s complex competitiveness framework cannot be measured directly, making it necessary to break it down into factors or pillars of manageable size as well as the necessary levels to reach a set of criteria or characteristics that can be measured [10]. Although they differ both in the number of countries covered and which factors determine the level of competitiveness, all of them include factors related to the macroeconomic environment, innovation and infrastructure.

It should also be noted that the Global Competitiveness Index (WEF-GCI), developed by the WEF, and the ranking included in the World Competitiveness Yearbook (WCY), which is issued by the IMD [11], combine hard statistical data provided by official agencies with others from executive opinion surveys (soft statistical data) given to business executives and/or entrepreneurs in the analyzed countries (IMD-WCY). Meanwhile, the European Competitiveness Index (CICUW-ECI), produced by the Centre for International Competitiveness from the University of Wales (UK), is based solely on quantitative data for a smaller set of variables (36 indicators grouped into three sub-indices or pillars) [12]. Similarly, the three indices differ in the way they aggregate the original variables to form the index; the first two use weighting that is established *a priori*. They also do not agree on which scale of scores to use: the WEF-GCI varies on a scale of 0–7; the scale of the index published in the WCY varies from 0–100, with 100 being the most competitive economy and 0 the least competitive, and lastly, the value of 100 in the ECI represents the average competitiveness of the European Union (EU-25) and the value of the index for a specific country reflects its deviation from this average value. In the year 2015, the WEF changes the composition of WEF-GCI. To understand and clarify the selection of years, this study is the first part developed during 2011–12 of a project for the period 2012–2020. The reader familiar with the topic of competitiveness will bear in mind the elaboration of the Global Sustainable Competitiveness Index. Developed in 2012 by a Korean consulting firm SolAbility [13], the Global Sustainable Competitiveness Index (Solability-GSCI) evaluates more than 180 countries and it is based on 127 measurable and comparable quantitative indicators in order to exclude the subjectiveness of qualitative indicators. ‘The Global Sustainable Competitiveness Report’ is published until today.

While the WEF-GCI methodology is geared towards the long term and therefore includes a wide variety of social and environmental indicators, which allows for a sustainable perspective of competitiveness in the analyzed countries, the methodology for the index produced by the IMD is oriented towards the short term, which consequently implies more frequent changes in the positions held by the different countries over time [14]. Additionally, the trend from 2012 years is to provide a competitiveness index for geographical clusters of countries: Europe, Latin America, Asia, among others.

There is no doubt that the two most influential and well-known indices out of the aforementioned indices are those developed by the WEF and the IMD. They have both received some criticism in the academic literature, especially the use of subjective data from opinion surveys given to experts in the analyzed countries [15], and the specification of the weights assigned to the basic criteria, which is also subjective [16].

In order to get around these problems, this paper proposes an alternative competitiveness index (CSI) based only on the objective data used by the WEF to create the WEF-GCI. This index, which is computed using a multidimensional statistical technique called Principal Component Analysis (PCA), allows for the objective assignment of weights for the basic criteria as well as the factors and pillars that eventually lead to the creation of said index. As a complement to the aforementioned, we have compared the rankings provided by both indices (CSI and WEF-GCI) and quantified the degree to which the proposed index (CSI) explains the variations of the WEF-GCI in the set of analyzed countries.

Following this introduction, Section 2 briefly describes the methodology used by the WEF to create the Global Competitiveness Index (WEF-GCI). Section 3 describes data and methodology, it explains the proposed synthetic index, including the methodology used to create it and an analysis of the obtained results. Section 4, concerning to the results and discussion, compares the rankings from the proposed index and the WEF-GCI on a global scale, using the scores to analyze the relationship between them in said context. Additionally, by limiting our study to the context of the European Union and to the WEF-GCI belongs to the 2007–08 period, we are able to compare the results obtained from the two aforementioned indices with the results obtained from the European Competitiveness Index *(*ECI) corresponding to the 2006–07 period (last available period). Developed by the Centre for International Competitiveness, the 2006–07 ECI report is the most recent report and this is the rationale for starting with the WEF-GCI of 2007–2008. Lastly, Sections 5 and 6 present the conclusions of this study and bibliographic references, respectively.

## 2. The global competitiveness Index produced by the WEF

Since 1979, the World Economic Forum has published the annual Global Competitiveness Report, whose main objective is to provide knowledge and stimulate debate among all the stakeholders about the best strategies and policies to help countries overcome obstacles to improve competitiveness [17]. The 2011–2012 Report defines competitiveness as "the set of institutions, policies and factors that determine the level of productivity a country." It also specifies that, in turn, the level of productivity establishes the level of prosperity an economy can achieve and also determines the rates of return obtained by investments, which are essential for growth [17]. Accordingly, within the context of this definition, competitiveness involves both static and dynamic components: productivity is identified as the determining factor of a country’s capacity to maintain its level of income as well as the investment returns, which is one of the basic factors in the explanation of an economy’s growth potential [18].

**Fig 1:**
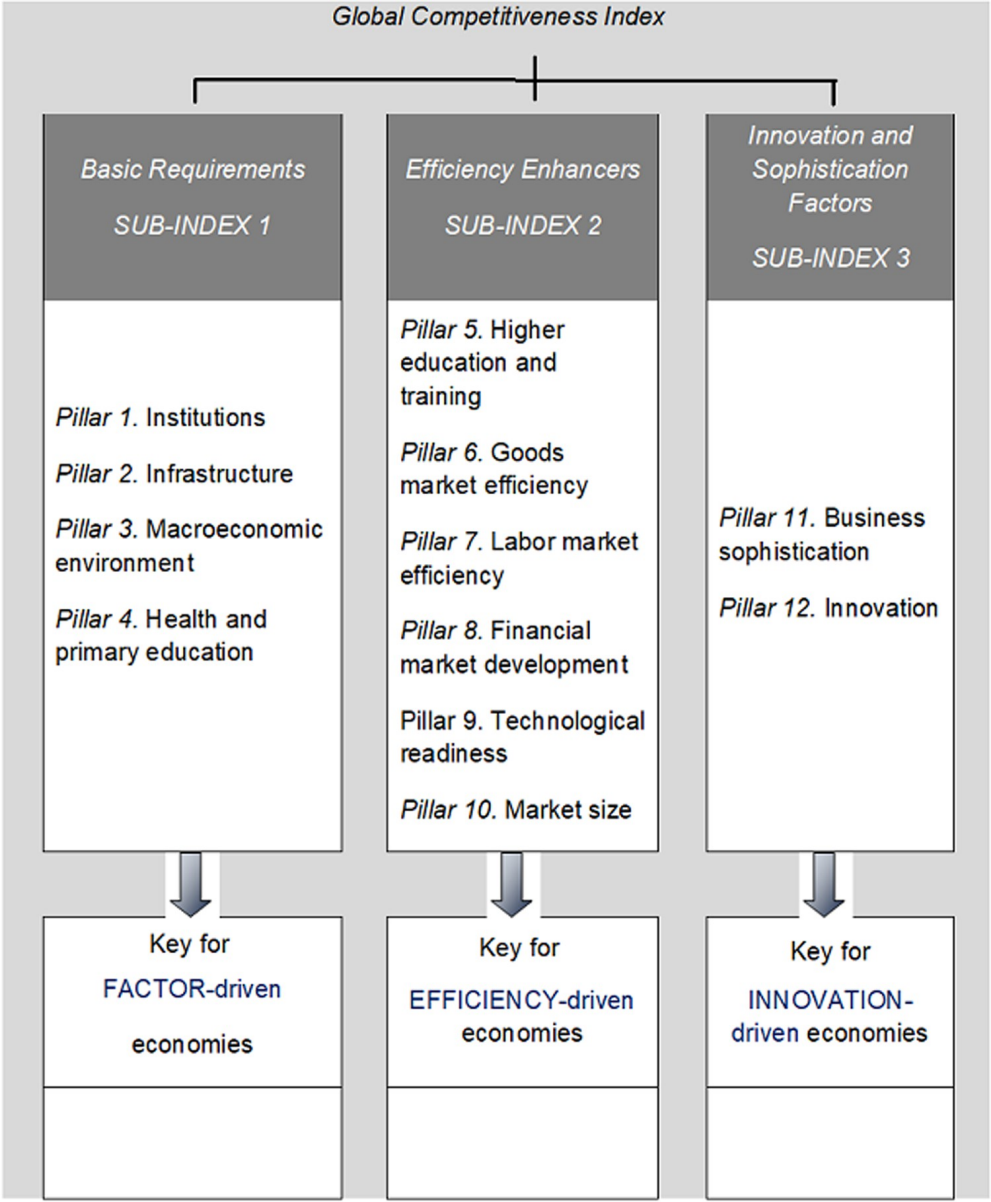
Groups of pillars in the global competitiveness index. Source: Fig 1 corresponds to a figure included, for example, in the 2010–11 Report [19, p. 9].

Since 2005, the WEF has based its analysis of competitiveness on the Global Competitiveness Index (WEF-GCI), which measures the microeconomic and macroeconomic basis of national competitiveness, according to the WEF [17]. Said index is supported by twelve basic pillars that are categorized into three factors or sub-indices: Basic Requirements; Efficiency Enhancers and Innovation and Sophistication (Fig 1 offers a more detailed configuration of WEF-GCI). The exact composition of the WEF-GCI and the technical details of its computation can be reviewed on the WEF website.

Each of the three sub-indices or factors has a different weight, which depends on the stage of development in each country. Accordingly, the criteria for determining the stage of development is unique and exclusively dependent on the GDP per capita, and the limits that determine the different groups of countries are arbitrary, considering their respective stage of development. Similarly, the weights assigned to the different sub-indices in each of ups are also arbitrary (see Table 1).

**Table 1:** Sub-index weights considering the stage of development.

	Stages of development				
	Stage 1: Factor-driven	Transition from 1 to 2	Stage 2: Efficiency-driven	Transition from 2 to 3	State 3: Innovation-driven
GDP p.c. (US $) Thresholds	<2000	2000–2999	3000–8999	9000–17000	>17000
Weight Sub-index 1	60%	40%-60%	40%	20%-40%	20%
Weight Sub-index 2	35%	35%-50%	50%	50%	50%
Weight Sub-index 3	5%	5%-10%	10%	10%-30%	30%

Source: [17].

Moreover, as previously mentioned, the concepts that are implicit in the pillars are not only quantified through statistical data published by internationally recognized official agencies (*hard* data), such as the United Nations Educational, Scientific and Cultural Organization (UNESCO), the International Monetary Fund (IMF) and the World Health Organization (WHO), but also with data from the annual opinion survey conducted by the WEF with executives from the analyzed countries (*soft* data).

Table 2 shows the total number of indicators used to quantify each pillar for the 2007-08 and 2010-11 periods. The Table 2 also presents the number of objective indicators that form each pillar (*hard* data) and their percentage share of the total indicators that form each pillar. Considering the data presented in Table 2, it is noteworthy that, for both periods, the characterization of four of the twelve pillars (Institutions, Financial Market Development, Business Sophistication, and Innovation) does not involve indicators considered to be *hard* data, as established in this paper, and that out of the total indicators used to compute the index, only approximately 25% is *hard* data, with the high subjectivity this entails.

**Table 2:** Number of indicators in each pillar and percentage of hard data.

	Period 2007–08			Period 2010–11		
Pillars	Indicators	Hard Data	% Hard Data/Total	Indicators	Hard Data	% Hard Data/Total
*Pillar 1*.*Institutions*	21	0	0.00	21	0	0.00
*Pillar 2*. *Infrastructure*	8	3	37.50	9	3	33.33
*Pillar 3*. *Macroeconomics environment*	4	4	100.00	5	4	80.00
*Pillar 4*. *Health and primary education*	10	6	60.00	10	6	60.00
*Pillar 5*. *Higher education and training*	8	2	25.00	8	2	25.00
*Pillar 6*. *Goods market efficiency*	15	5	33.33	15	5	33.33
*Pillar 7*. *Labor market efficiency*	7	2	28.57	7	2	28.57
*Pillar 8*. *Financial market development*	6	0	0.00	8	0	0.00
*Pillar 9*. *Technological readiness*	5	2	40.00	5	2	40.00
*Pillar 10*. *Market size*	4	2	50.00	4	2	50.00
*Pillar 11*. *Business sophistication*	9	0	0.00	9	0	0.00
*Pillar 12*. *Innovation*	6	0	0.00	6	0	0.00
*Total of indicators*	*103*	*26*	*25*.*24*	*107*	*26*	*24*.*30*

Source: Own elaboration from data included in WEF [17].

## 3. Data and methodology

The indicators used and their names for reference in future tables are listed in Table 3 and are based on most of the objective, quantitative data (hard data) from the 2007–2008 and 2010–2011 periods, which is available on the WEF website (http://www.weforum.org/).

**Table 3:** Indicators used to compute CSI.

Pillar	Description		Pillar
2	Mobile telephone subscriptions /100 inhabitants	X_1_	Pillar2X1
2	Telephone land lines /100 inhabitants	X_2_	Pillar2X2
3	Government budget balance (% of GDP)	X_13_	Pillar3X1
3	Gross National Savings (% of GDP)	X_14_	Pillar3X2
3	Inflation (inter-annual variability %)	X_19_	Pillar3X3
3	Government debt (% of GDP)	X_17_	Pillar3X4
4	Cases of malaria /100,000 inhabitants	X_9_	Pillar4X1
4	Cases of tuberculosis /100,000 inhabitants	X_6_	Pillar4X2
4	Prevalence of HIV-AIDS (% of the adult population)	X_7_	Pillar4X3
4	Infant mortality rate /1,000 births	X_8_	Pillar4X4
4	Life expectancy (years)	X_5_	Pillar4X5
4	Enrollment in primary education (% net)	X_10_	Pillar5X1
5	Enrollment in secondary education (% gross)	X_11_	Pillar5X2
5	Enrollment in higher education (% gross)	X_12_	Pillar5X3
6	Total tax rate (%)	X_18_	Pillar6X1
6	Number of procedures required to start a business	X_20_	Pillar6X2
6	Number of days required to start a business	X_21_	Pillar6X3
6	Imports (% of GDP)	X_22_	Pillar6X4
7	Cost of layoffs (weeks of salary)	X_16_	Pillar7X1
7	Women’s participation in the labor market (ratio in regard to men)	X_15_	Pillar7X2
9	Individuals that use the Internet (%)	X_3_	Pillar9X1
9	Fixed broadband Internet subscriptions /100 inhabitants	X_4_	Pillar9X2
10	Exports (% of GDP)	X_23_	Pillar10X1
Ancillary	GDP (billions of US dollars)*	X_26_	Anc1
Ancillary	GDP per capita (US dollars)*	X_25_	Anc2
Ancillary	Population (millions of people)*	X_24_	Anc3

Source: Prepared by the authors. Note

* = Available in the WEF database for 2010–11.

It is shown that some pillars do not consider hard data indicators. Of the total number of countries included in the WEF database, this study only involves 79, which are the countries that have complete data available corresponding to the variables included in Table 3 for the two periods under consideration.

We used the Exploratory Factor Analysis (EFA) multivariate technique to compute the proposed synthetic competitiveness indicator (CSI). This exploratory technique applies to the multidimensional data analysis of a set of elements that are described through a high number of quantitative variables and allows us to synthesize the data regarding said variables in a reduced number of latent variables or factors, which aim to explain the reality of said elements (in this case, the set of analyzed countries) in relation to the complex characteristic being studied (in this case, their competitiveness).

Since the results of the EFA are later used to compute the synthetic competitiveness indicator, which requires the factor scores, we used the Principal Component Analysis extraction method [20], retaining the factors with a higher eigenvalue than the unit [21]. The *varimax* orthogonal rotation procedure was applied in order to obtain the interpretation of the retained factors*)* [22].

Finally, we used the scores of the retained factors to create the synthetic competitiveness indicator (CSI) as a weighted average of said scores. These weights are given by the percentage of the total variance explained by each factor identified by the EFA.

## 4. Results of the exploratory factor analysis

The determinant values of the correlation matrices for the 2007-08 and 2010-11 periods, (1.02·10^−11^ and 1.18·10^−11^, respectively), very close to zero, indicate the existence of linear dependence between the indicators included in this study for both of these periods and the non-existence of redundant indicators; in other words, they are the perfect linear combination of others that are also included in the analysis. Based on these determinants, we have calculated the values of the effective dependence coefficients associated with them, D_07-08_(R) = 0.6366 and D_10-11_(R) = 0.6345, which indicate the existence of a considerable degree of linear dependence between the variables involved in each of the analyzed periods. This coefficient is defined as D(R) = 1-|R|1/(P-1) where |R| is the determinant of the correlations. We know that if one of the variables is the perfect linear combination of others, which are also included in the analysis, the correlation matrix is singular, |R| = 0, and therefore, D(R) = 1. Meanwhile, if the linear correlation between different pairs of variables is null, the correlation matrix coincides with the identity, its determinant is 1 and, therefore, D(R) = 0. The comparison of the dependence coefficient with these extreme cases can give us a good idea of the degree of linear dependence between the indicators used [23].

As a complement to the linear dependence analysis, we calculated the KMO index (Kaiser-Meyer-Olkin). For this analysis, the values of the KMO index are 0.824 and 0.790, respectively. They are both higher than the minimum recommended value for this type of study (0.5) and, therefore, the application of this methodology is considered to be acceptable. According to the factor analysis model, the theoretical correlation coefficients calculated between each pair of unique factors are null by hypothesis. If the partial correlation coefficients approach said theoretical coefficients, they must be close to zero and, therefore, the value of the KMO index should be close to 1. Values of the KMO measurement below 0.5 are not acceptable [24].

Table 4 includes the eigenvalues associated with the retained factors, the percentage of the total explained variance for each of these factors after the varimax rotation and the accumulated percentage. This table shows that competitiveness can be synthesized in seven factors for the two analyzed periods, according to the criteria based on selecting the factors associated with higher eigenvalues than the unit [21]. These factors explain 77% and 76.5% of the total variability, an acceptable percentage considering that the lower limit of acceptability for studies in the social sciences is 60% [25].

**Table 4:** Total explained variance. Principal component analysis.

	*Eingen values*			*Extraction Sums of Squares Loadings*			*Rotation Sums of Squares Loadings*		
	Total	% of the variance	% accumulated	Total	% of the Variance	% accumulated	Total	% of the Variance	% accumulated
*Components*	*Period 2007–2008*								
C1	9.515	36.598	36.598	9.515	36.598	36.598	7.395	28.441	28.441
C2	2.710	10.424	47.022	2.710	10.424	47.022	3.122	12.007	40.448
C3	2.244	8.633	55.655	2.244	8.633	55.655	2.483	9.550	49.999
C4	1.844	7.094	62.748	1.844	7.094	62.748	2.281	8.772	58.771
C5	1.460	5.614	68.362	1.460	5.614	68.362	1.852	7.122	65.892
C6	1.301	5.002	73.365	1.301	5.002	73.365	1.576	6.061	71.953
C7	1.039	3.997	77.362	1.039	3.997	77.362	1.406	5.409	77.362
C8	.829	3.188	80.550						
C9	.789	3.035	83.585						
C10	.721	2.775	86.360						
C11	.569	2.190	88.550						
C12	.484	1.860	90.410						
C13	.471	1.812	92.222						
C14	.384	1.475	93.697						
C15	.311	1.195	94.893						
C16	.280	1.079	95.971						
C17	.238	.916	96.888						
C18	.177	.681	97.568						
C19	.143	.550	98.118						
C20	.126	.485	98.603						
C21	.086	.330	98.933						
C22	.081	.311	99.244						
C23	.073	.282	99.526						
C24	.057	.218	99.745						
C25	.038	.147	99.892						
C26	.028	.108	100.000						
*Components*	*Period 2010–11*								
C1	9.129	35.110	35.110	9.129	35.110	35.110	8.177	31.451	31.451
C2	2.931	11.274	46.384	2.931	11.274	46.384	2.525	9.712	41.163
C3	2.194	8.437	54.821	2.194	8.437	54.821	2.304	8.860	50.023
C4	1.666	6.409	61.230	1.666	6.409	61.230	2.143	8.241	58.264
C5	1.567	6.027	67.257	1.567	6.027	67.257	1.785	6.864	65.127
C6	1.396	5.369	72.627	1.396	5.369	72.627	1.671	6.428	71.556
C7	1.004	3.863	76.490	1.004	3.863	76.490	1.283	4.934	76.490
C8	.949	3.652	80.142						
C9	.758	2.916	83.057						
C10	.697	2.681	85.738						
C11	.635	2.444	88.182						
C12	.602	2.316	90.498						
C13	.466	1.792	92.290						
C14	.395	1.519	93.809						
C15	.286	1.101	94.910						
C16	.247	.949	95.859						
C17	.201	.775	96.634						
C18	.195	.749	97.383						
C19	.156	.599	97.982						
C20	.140	.539	98.521						
C21	.124	.477	98.998						
C22	.087	.336	99.334						
C23	.073	.279	99.613						
C24	.047	.179	99.793						
C25	.032	.123	99.916						
C26	.022	.084	100.000						

Source: Prepared by the authors using SPSS.

Table 5 shows the factor loading matrices after the varimax rotation or rotated component matrices, which are formed by the linear correlation coefficients between the factors and the indicators used to estimate them. In order to facilitate the interpretation of said factors, in terms of the different aspects related to the competitiveness of said factors, the coefficients that have an absolute value greater than 0.4 are shaded in a darker color.

**Table 5:** Matrix of rotated components. Rotation method: Varimax.

*INDICATORS*	*Components (2007–08)*							*Components (2010–11)*						
	*C1*	*C2*	*C3*	*C4*	*C5*	*C6*	*C7*	*C1*	*C2*	*C3*	*C4*	*C5*	*C6*	*C7*
Pillar2_X1	.826	.196	.232	.110	.051	-.077	.027	.744	.026	.142	.183	.075	-.242	-.017
Pillar2_X2	.884	.156	.254	.017	-.029	.113	.150	.863	.202	.017	-.067	-.218	.122	-.052
Pillar3_X1	.833	.094	.357	.026	-.003	.032	.174	.876	.323	.081	-.039	-.096	.040	.080
Pillar3_X2	.818	.001	.360	.054	.011	.066	.183	.833	.377	-.007	-.162	-.153	.092	.000
Pilar3_X3	.699	.637	.116	.044	.058	.032	-.086	.837	.129	.045	.446	-.046	.040	-.072
Pillar3_X4	.396	.821	.215	-.036	.025	.026	-.022	.555	.218	-.114	.662	-.105	-.036	-.165
Pillar4_X1	.132	.867	.101	-.070	-.059	.044	.080	.374	.186	-.131	.763	-.042	-.001	-.107
Pillar4_X2	.765	.449	.050	.116	.121	-.030	-.099	.905	-.001	.141	.259	.030	.018	.022
Pillar4_X3	.344	.667	-.131	.094	.029	.015	-.082	.611	-.279	.259	.272	.229	.137	.187
Pillar4_X4	.674	.239	-.200	.153	-.100	-.015	-.229	.583	-.021	.060	.007	-.001	.040	-.535
Pillar4_X5	.778	.371	.076	.087	.150	-.032	.104	.885	.074	.048	.042	.027	-.012	.059
Pillar5X1	.814	.288	.148	-.082	.036	-.038	.269	.877	.023	-.100	.044	-.171	-.047	.088
Pillar5X2	.289	.044	.038	.007	.799	-.145	.034	-.066	.236	-.043	-.020	.808	-.030	-.165
Pillar5X3	.032	-.099	-.165	.415	.705	.322	-.148	.003	-.098	.398	.031	.651	.426	.064
Pillar6X1	.432	-.186	-.154	-.076	-.005	-.002	.708	.364	.206	-.165	-.675	-.046	-.061	-.077
Pillar6X2	.079	.176	.410	.161	.142	-.021	.649	.347	.232	-.024	-.025	-.083	-.012	.598
Pillar6X3	-.206	.006	.055	-.366	.732	-.155	.213	-.329	-.223	-.305	-.083	.639	-.218	.139
Pillar6X4	.080	-.383	.380	.178	.203	-.405	-.203	-.078	.097	.354	-.384	.007	-.154	.449
Pillar7X1	.383	.064	.598	.107	.064	.177	-.147	.277	.669	.118	.125	.127	.048	-.219
Pillar7X2	.424	-.119	.708	.093	-.047	-.151	.208	.185	.745	.141	-.179	.043	-.167	.405
Pillar9X1	.237	.198	.735	-.008	-.069	.016	.083	.102	.774	.133	.102	-.038	.011	.220
Pillar9X2	.020	-.072	.090	.941	-.128	-.159	.056	-.006	.146	.919	-.050	-.110	-.137	.049
Pillar10X1	.146	.068	.086	.937	.091	-.099	.010	.234	.156	.873	.006	.064	-.117	-.021
Anc1	-.132	.060	-.056	-.042	.027	.847	-.116	-.122	-.134	-.062	.099	.131	.859	-.112
Anc2	.807	.023	.366	-.011	.085	.065	.144	.759	.383	-.011	-.166	-.138	.123	.071
Anc3	.284	-.023	.176	-.202	-.127	.648	.110	.218	.148	-.273	-.053	-.183	.699	.016

Source: Prepared from authors using SPSS.

In regard to the interpretation of the retained factors, it should be noted that the only factor that is common to both periods (C5) is the one that includes the variables related to the macroeconomic environment (Pillar 3), with the exception of inflation (Pillar3X3). Said factor explains approximately 7% and can be called “*Government budget balance*, *savings and debt*.*”*

Meanwhile, other factors related to the 2007–08 period, such as C3, C4, and C6, could be identified in 2010–11 with C2, C3, and C6, respectively, since the variables that have higher correlations with each of these factors are the same for both periods. Accordingly, C3 (2007–08) has high correlations with the variables that quantify inflation (Pillar3X3) and the number of days and procedures required to start a business (Pillar6X3). The loading for this factor, C3, for the 2007–08 period also includes the variable that quantifies the costs of layoffs (Pillar 7), although it is less significant than those previously mentioned. Therefore, we call this factor “*Inflation and ease of doing business*,” which is identified with C2 for the 2010–11 period and explains 9.6% (9.7%) of the total variability. Similarly, C4 (2007–08) can be called “*Foreign trade*,” since the higher correlations correspond to the percentage of imports (Pillar6X4) and exports (Pillar10X1) in the GDP. This factor is identified with C3 (2010–11) and explains 8.8% (8.9%) of the total variability. Lastly, the variables that quantify the number of the country’s inhabitants and income (both ancillary, Anc3 and Anc2, GDP per capita as a proxy of citizen’s income) are included in C6 for both periods. This factor, C6, which we call “*Overall country size*,” explains 6.1% (6.4%) of the total variability. Note it presents a weak, negative correlation with the normalized variable that quantifies the total tax rate (Pillar6X1) for the 2007–08 period, while it appears to be correlated with the Gross National Savings for the 2010–11 period (Pillar3X2).

Theoretically, we cannot establish as clear of a correspondence for the remaining factors in the 2007–08 period and the 2010–11 period as those described in the previous paragraph. However, C1 presents intense correlations with the variables related to infrastructure (Pillar 2), innovation (Pillar 9), education (Pillars 4 and 5), per capita income (Anc2) and some health-related variables (infant mortality, Pillar4X4, and life expectancy, Pillar4X5) for both periods. This factor also appears to be associated with other health variables, cases of malaria (Pillar4X1) and tuberculosis (Pillar4X2) for the 2010–11 period. It presents a weak correlation with the women’s participation in the labor market (Pillar7X2) for the 2007–08 period and the number of procedures required to start a business (Pillar6X2). According to the structure of said factor (which is common to both periods), a possible name could be “*Human development and ease of communication*.” This factor explains 28.4% (31.5%) of the total variability.

Meanwhile, the C2 factor (12% of the total variability) for the 2007–08 period unites all of the variables that quantify health-related aspects, even those that appear to be accounted for in Factor C1. However, this factor is not exclusive of health for the 2010–11 period, but some of the variables included in this factor (such as life expectancy and cases of tuberculosis and HIV) are correlated with C4 (8.2% of total variability). It is surprising that said component presents a high negative correlation with the variable that quantifies women’s participation in the labor market (Pillar7X2). According to the above, Factor C2 (2007–08) could be called “*Health*,” but we cannot find an appropriate name or clear interpretation for Factor C4 (2010–11).

The last component (C7) appears to be associated with the labor market (Pillar 7) for the 2007–08 period, both in terms of the cost of layoffs (Pillar7X1) and women’s participation in the labor market (Pillar7X2). The name (“*Labor market*”) is clear and explains 5.4% of the total variability. For the 2010–11 period, this component presents moderate, positive levels of correlation with the variables that quantify the cost of layoffs, the total tax rate (Pillars6X1) and the number of procedures required to start a business (Pillars6X2), as well as a moderate, negative correlation with the participation in primary education (Pillar5X1). This factor could complement the data on “ease of doing business” provided by Factor C2 for the same period, but the negative correlation that said factor has with participation in primary education makes its interpretation more complicated.

Table 6 presents a summary of the names of the factors identified in the two periods under consideration, their identification with the corresponding component, and the pillars that encompass the variables that allow for their interpretation.

**Table 6:** Names of factors, components and pillars.

Factor names	2007–08	2010–11	Pillars
*Human development and ease of communication*	C1	C1^(a)^	2, 4, 5, 9 and ancillary
*Health*	C2		4
*Inflation and ease of doing business*	C3^(b)^	C2	3 and 6
*Foreign trade*	C4^(c)^	C3	6 and 10
*Government budget balance*, *savings and debt*	C5	C5	3
*Overall country size*	C6	C6^(d)^	Ancillary
*Labor market*	C7		7
*Complement to C1 in health and women’s participation in the labor market*		C4	4 and 7
*Complement to C2 in ease of doing business*		C7	4, 6 and 7

Note.

(a) 2010–11 also includes cases of malaria and tuberculosis.

(b) 2007–08 also presents a correlation close to 0.4 with the costs of layoffs.

(c) 2007–08 also presents a correlation close to 0.4 with the Gross National Savings.

(d) 2010–11 also presents a correlation close to 0.4 with the Gross National Savings.

Source: Prepared by authors using SPSS.

The data provided by the scores of the retained factors have been synthesized in a sole index, the synthetic competitiveness index (CSI), which summarizes the situation of each of the analyzed countries in terms of competitiveness. Said indicator was obtained as a weighted average of the scores for the seven retained factors, using the percentage of the total variance explained by each factor as the weight of the score for each factor.

It should bear in mind other relevant factors not considered in this WEF indicators, so, these missed relevant factor are given 0% of the total explained variance. Be aware, there is not included hard data into the following pillars: the Pillar 1 –Institutions–, the Pillar 2 –Financial market development–, the Pillar 11 –Business sophistication–and the Pillar 12–Innovation–.

The following section presents an analysis of the results of said indicator for the two analyzed periods.

## 4. Results and discussion

### 4.1. Rankings and geographic distributions: WEF-GCI versus CSI

This section presents a comparison of the rankings provided by the proposed index (CSI) and the WEF-GCI, on a global scale. Table 7 shows the rankings from the CSI and WEF-GCI for the analyzed countries and periods.

**Table 7:** Rankings and geographic distributions: WEF-GCI versus CSI.

	2007–08			2010–11			2007–08			2010–11	
Country	GCI	CSI	Country	GCI	CSI	Country	GCI	CSI	Country	GCI	CSI
United States	1	3	Switzerland	1	4	Mauritius	41	37	Brazil	41	61
Switzerland	2	7	Sweden	2	5	Kazakhstan	42	43	Vietnam	42	51
Denmark	3	4	Singapore	3	1	Costa Rica	43	38	Russian Federation	43	38
Sweden	4	6	United States	4	13	Morocco	44	62	Uruguay	44	41
Germany	5	17	Germany	5	15	Greece	45	34	Mexico	45	39
Finland	6	8	Finland	6	10	Azerbaijan	46	42	Colombia	46	48
Singapore	7	1	Netherlands	7	6	El Salvador	47	61	Latvia	47	27
United Kingdom	8	16	Denmark	8	3	Vietnam	48	48	Kazakhstan	48	37
Netherlands	9	5	Canada	9	16	Colombia	49	49	Peru	49	49
Canada	10	11	United Kingdom	10	21	Brazil	50	53	Namibia	50	69
Taiwan, China	11	14	Taiwan, China	11	17	Ukraine	51	41	Morocco	51	45
Austria	12	19	Norway	12	2	Uruguay	52	44	Guatemala	52	66
Norway	13	2	France	13	19	Algeria	53	32	El Salvador	53	58
Israel	14	25	Australia	14	7	Honduras	54	55	Greece	54	42
France	15	18	Austria	15	14	Trinidad and Tobago	55	36	Trinidad and Tobago	55	44
Australia	16	12	Belgium	16	8	Argentina	56	45	Algeria	56	52
Belgium	17	9	New Zealand	17	9	Peru	57	50	Argentina	57	46
Malaysia	18	30	Israel	18	31	Guatemala	58	68	Ukraine	58	54
Ireland	19	13	Malaysia	19	32	Namibia	59	70	Honduras	59	65
Iceland	20	10	China	20	20	Georgia	60	52	Georgia	60	50
New Zealand	21	15	Ireland	21	18	Pakistan	61	75	Armenia	61	47
Chile	22	35	Chile	22	35	Armenia	62	51	Dominican Republic	62	62
Estonia	23	20	Iceland	23	26	Dominican Republic	63	63	Benin	63	75
Spain	24	23	Tunisia	24	36	Venezuela	64	54	Senegal	64	70
Tunisia	25	46	Estonia	25	12	Kenya	65	76	Ecuador	65	60
Czech Republic	26	24	Oman	26	11	Senegal	66	72	Kenya	66	76
China	27	22	Czech Republic	27	25	Ecuador	67	58	Bolivia	67	67
Lithuania	28	26	Poland	28	33	Tanzania	68	77	Cambodia	68	73
Portugal	29	33	Spain	29	28	Bolivia	69	56	Guyana	69	63
Oman	30	39	Indonesia	30	56	Benin	70	73	Nicaragua	70	59
South Africa	31	67	Portugal	31	40	Cambodia	71	71	Tanzania	71	77
Latvia	32	27	Lithuania	32	34	Nicaragua	72	65	Zambia	72	79
Italy	33	21	Italy	33	22	Burkina Faso	73	78	Paraguay	73	57
Hungary	34	29	India	34	55	Madagascar	74	74	Kyrgyz Republic	74	43
India	35	57	Hungary	35	23	Kyrgyz Republic	75	60	Venezuela	75	64
Poland	36	31	Panama	36	30	Paraguay	76	64	Pakistan	76	72
Mexico	37	47	South Africa	37	71	Zambia	77	79	Madagascar	77	68
Indonesia	38	66	Mauritius	38	29	Lesotho	78	69	Lesotho	78	74
Russian Federation	39	28	Costa Rica	39	53	Guyana	79	59	Burkina Faso	79	78
Panama	40	40	Azerbaijan	40	24						

Source: Prepared by the authors, results obtained using ArcGIS.

We use the scores to analyze the geographic distribution of the indices within said context and the relationship between the two. Additionally, limiting our study to countries in the European Union, we have compared the results obtained for the 2007–08 period for the two aforementioned indices with those from the European Competitiveness Index (ECI) for the 2006–07 period.

Considering the classification presented in Table 7, according to the CSI, we can see that the most competitive countries for the two analyzed periods are Singapore and Norway. Similarly, although it may seem obvious, the two indices provide different rankings, both in terms of the majority of the countries that are classified as the most competitive according to the WEF-GCI (the first 20 countries) and the majority of the countries that are classified as the least competitive (the last 20 countries), which remain in the same group according to the alternative index CSI.

Meanwhile, based on the rankings shown in Table 7, the correlation coefficients have been calculated according to Spearman’s rank correlation corresponding to the rankings provided by both indices in Table 8. This coefficient quantifies the degree of association between the two rankings and indicates their direction, as well as the association between the WEF-GCI for the countries analyzed in this study, which is statistically significant, positive and high.

**Table 8:** Spearman’s rho: Correlation between the WEF-GCI and CSI.

			2007–08		2010–11	
			WEF-GCI	CSI	WEF-GCI	CSI
Spearman’s rho	**GCI**	Correlation Coefficient	1.000	.884**	1.000	.884**
		Sig. (2-tailed)	.	.000	.	.000
		Total countries (N)	79	79	79	79
	**CSI**	Correlation Coefficient	.884**	1.000	.884**	1.000
		Sig. (2-tailed)	.000	.	.000	.
		Total countries (N)	79	79	79	79

**. Correlation is significant at the 0,01 level (2-tailed).

Source: Prepared by the authors, results obtained using SPSS.

Table 9 presents a summary of the countries that are among the least competitive (first quintile) and the most competitive (last quintile), respectively, for the two analyzed periods, according to both indices.

**Table 9:** Summary of the most and least competitive countries: WEF-GCI versus CSI.

TRADITIONAL CONTINENTAL MODEL (UN)	1^st^ QUINTILE: COUNTRIES IN COMMON WEF-GCI AND CSI. 2007–08	1^st^ QUINTILE: COUNTRIES IN COMMON WEF-GCI AND CSI. 2010–11
*AMERICA*	Nicaragua, Paraguay	Bolivia
*AFRICA*	Benin, Burkina Faso, Kenya, Lesotho, Madagascar, Senegal, Tanzania, Zambia	Burkina Faso, Guyana, Kenya, Lesotho, Madagascar, Senegal, Tanzania, Zambia
*EUROPA*		
*ASIA*	Cambodia	Cambodia, Pakistan
*OCEANÍA*		
**TRADITIONAL CONTINENTAL MODEL (UN)**	**5**^**th**^ **QUINTILE: COUNTRIES IN COMMON WEF-GCI AND CSI. 2007–08**	**5**^**th**^ **QUINTILE: COUNTRIES IN COMMON WEF-GCI AND CSI. 2010–11**
*AMERICA*	Canada, United States	United States
*EUROPA*	Denmark, Finland, Netherlands, Norway, Sweden, Switzerland	Austria, Denmark, Finland, Germany, Netherlands, Norway, Sweden, Switzerland
*ASIA*	Singapore	Singapore
*OCEANÍA*	Australia	Australia

Source: Prepared by the authors.

**Fig 2:**
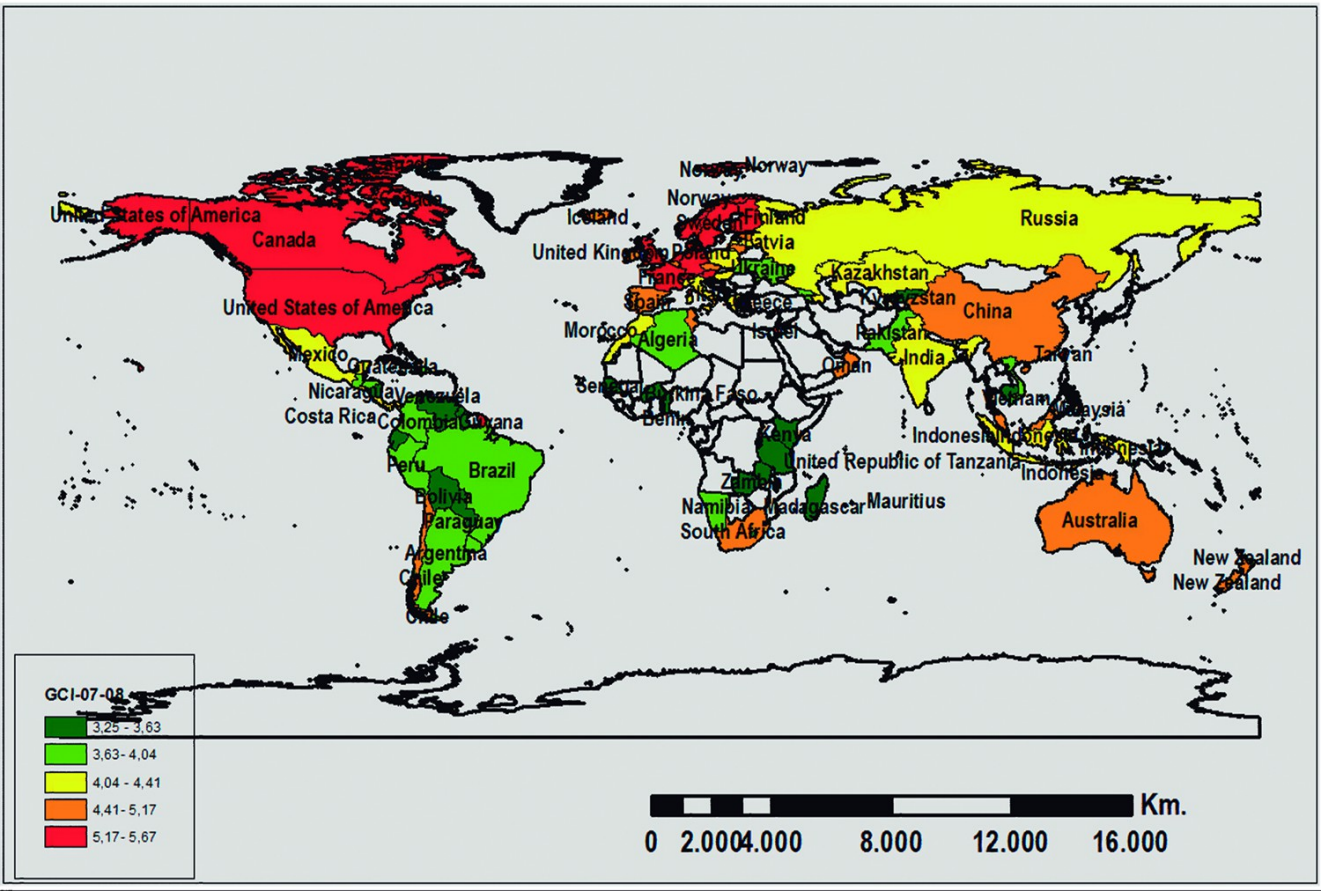
Geographic distribution of the WEF-GCI scores for 2007–08. Source: Prepared by authors using ArcGIS software.

**Fig 3:**
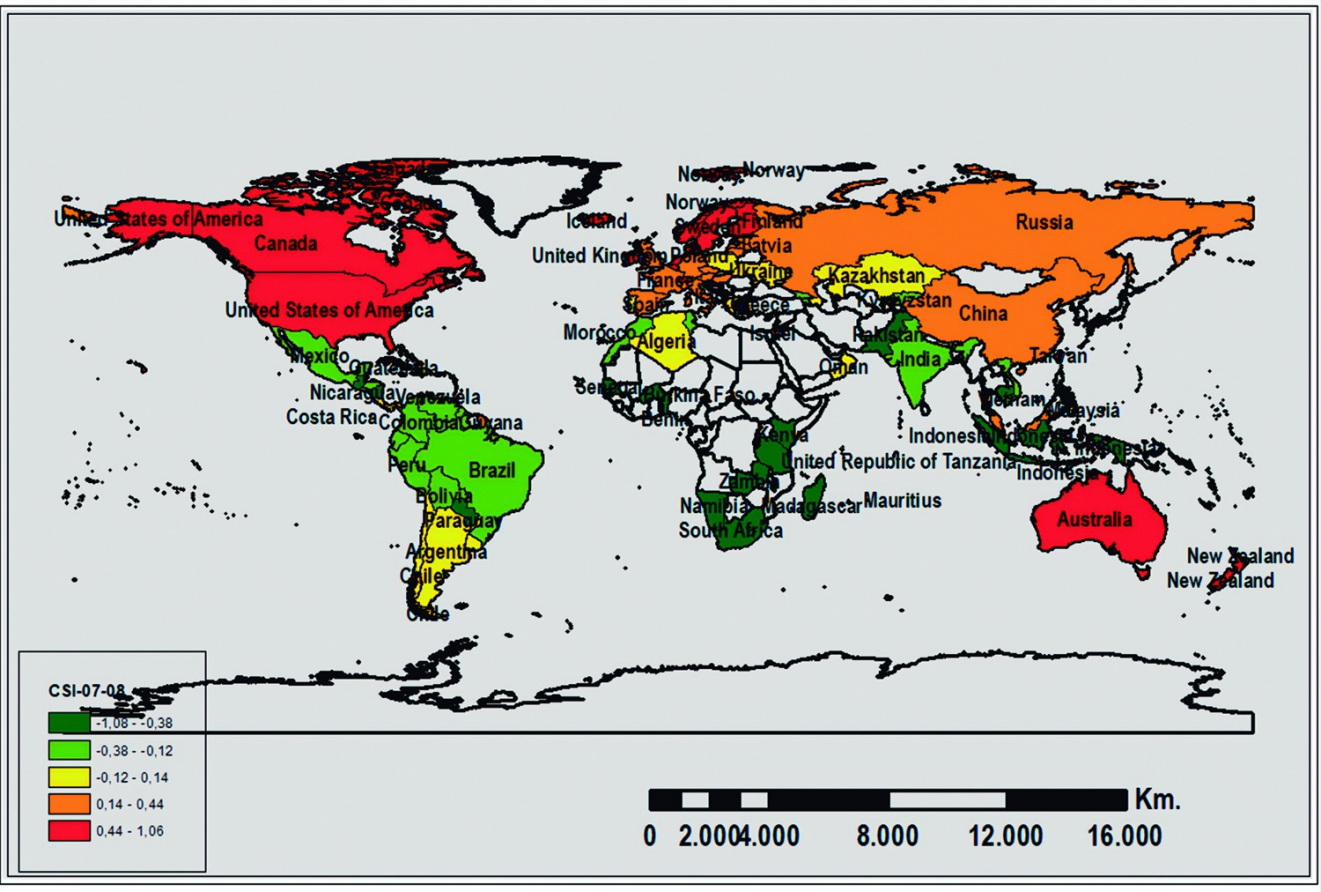
Geographic distribution of the CSI scores for 2007–08. Source: Prepared by authors using ArcGIS software.

**Fig 4:**
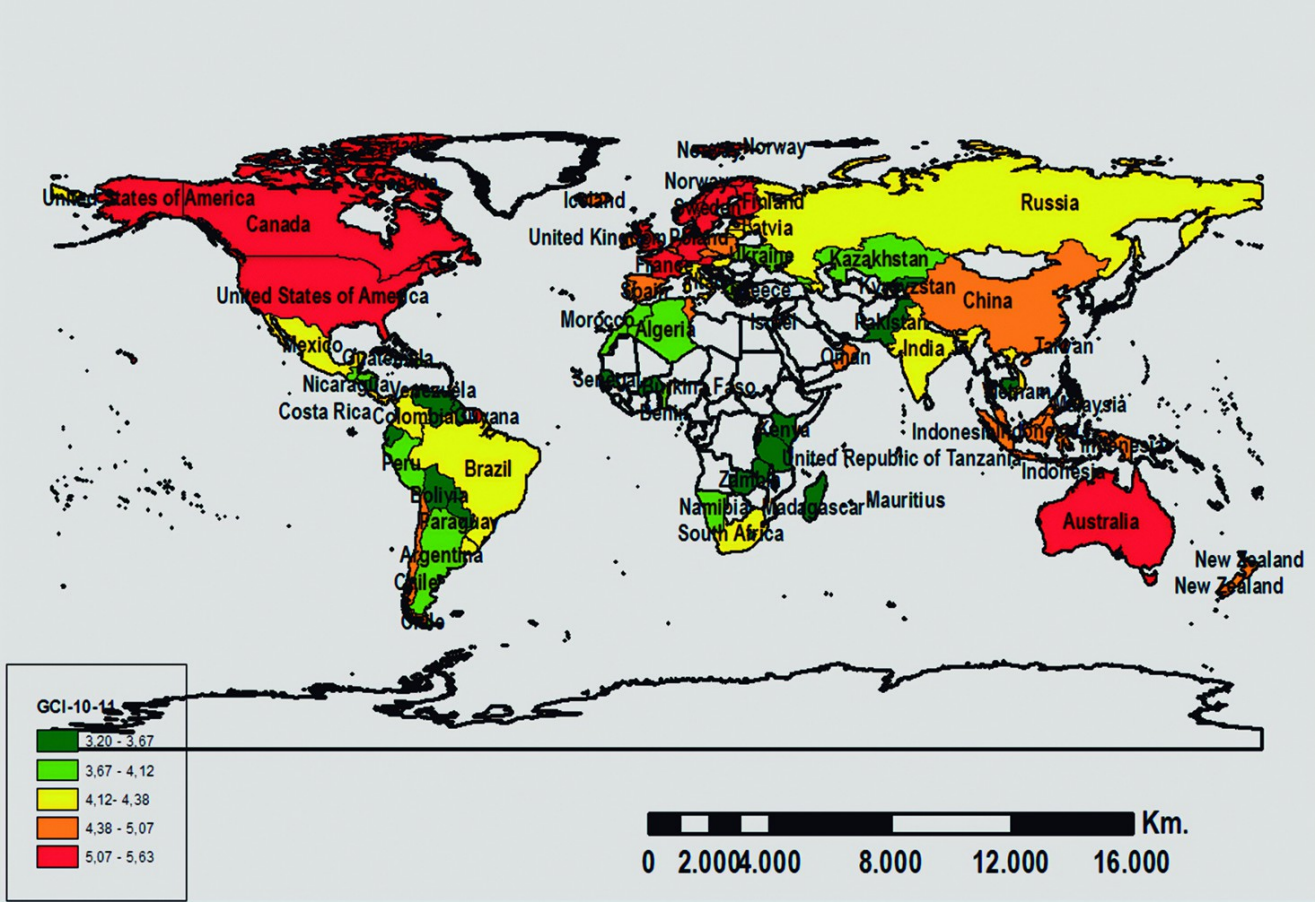
Geographic distribution of the WEF-GCI scores for 2010–11. Source: Prepared by authors using ArcGIS software.

**Fig 5:**
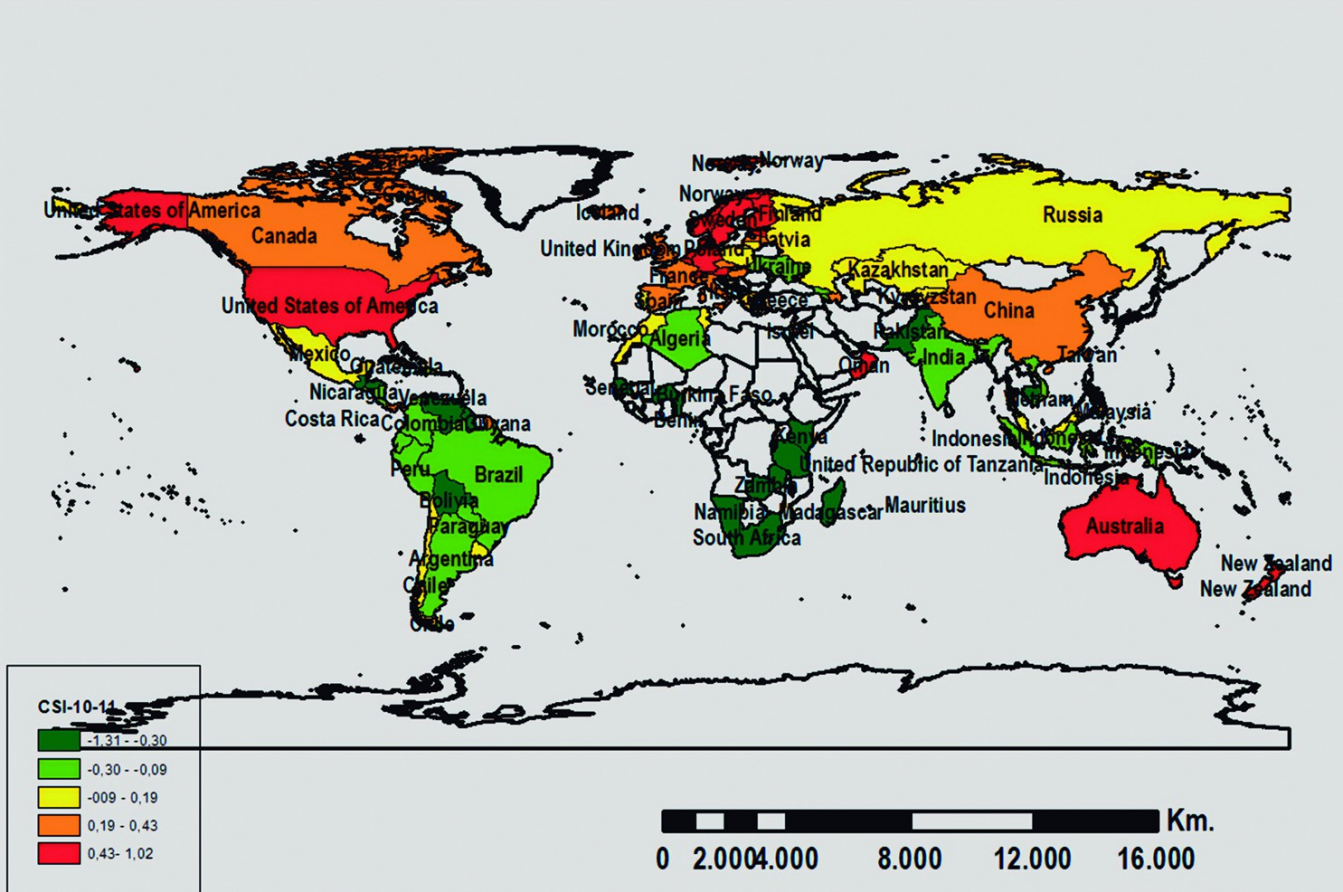
Geographic distribution of the CSI scores for 2010–11. Source: Prepared by authors using ArcGIS software.

In turn, the country distribution considering their scores in the GCI and CSI for the two analyzed periods is shown in Figs 2, 3, 4, and 5. The geographic representation is based on the quintiles (values that divide the corresponding distribution into five types, each with the same number of countries, approximately).

In summary, and in terms of the evolution of the countries positioned in the first and fifth quintiles, it is noteworthy that for both periods, the majority of the least competitive countries are located in Africa, except for Cambodia, while the most competitive countries are located in Australia (Oceania), United States (America), Singapore (Asia), and others in Central and Northern Europe (Denmark, Finland, Holland, Norway, Sweden, and Switzerland). According to Loo (2012), Singapore was ranked 1st in the rank using an average between IMD-WCY and WEF-GCI. Moreover, the 2012 GSCI report [13] indicates that countries in northern Europe are the leading countries: Denmark–rank 1–, Sweden–rank 2– Norway–rank 3– have the highest rakings, although this index is proposed from a sustainable perspective.

Furthermore, in terms of the evolution of the WEF-GCI over time (see Figs 2 and 4), it should be noted that the composition of the first quintile for the two time periods is very similar and over 50% of the countries classified in this quintile are on the African continent. Similarly, all of the countries classified in the last quintile for the 2007–08 period, except for Israel, remain in the same quintile for the 2010–11 period. The most competitive countries include the United States (America), China, Taiwan (China), and Singapore (Asia), as well as numerous countries in Central and Northern Europe (Germany, Austria, Denmark, Finland, France, Holland, Norway, United Kingdom, Sweden, and Switzerland).

Finally, the CSI shows a very similar evolution as mentioned in the previous paragraph (see Figs 3 and 5). Accordingly, the majority of the least competitive countries maintain their position in the two analyzed periods and are generally African countries. Meanwhile, the most competitive countries for both periods belong to Oceania (Australia), America (United States), Central and Northern Europe (Denmark, Finland, Holland, Norway, Sweden, and Switzerland), and Asia (Singapore). Furthermore, there is a group of countries that, relatively speaking, experienced a decline in the 2010–11 period as compared to the 2007–08 period: Canada (America), Ireland, Iceland (Europe), and Taiwan (Asia). At the same time, other countries’ relative competitiveness improved from one period to the next: Austria, Germany, Estonia (Europe), and Oman (Asia).

### 4.2. Competitiveness in Europe: WEF-GCI, CICUW-ECI and CSI

In terms of the competitiveness of European countries for the 2007–08 period, as previously mentioned, this section compares the results obtained for the two aforementioned indicators with those corresponding to the European Competitiveness Index (ECI) for the 2006–07 period. This comparison is based on the data in common among 22 countries: Germany, Austria, Belgium, Denmark, Spain, Estonia, Finland, France, Greece, Holland, Hungary, Ireland, Italy, Latvia, Lithuania, Norway, Poland, Portugal, United Kingdom, Czech Republic, Sweden, Switzerland. The association between the rankings based on the scores of said indices, measured by Spearman’s correlation coefficient, is considered to be statistically significant, positive, and high for the different pairs considered in this study, as shown in Table 10.

**Table 10:** Spearman’s correlations between ranks for the GCI and CSI in Europe.

			2006–07	2007–08	
			ECI	GCI	CSI
Spearman’s rho	**ECI**	Correlation Coefficient	1	.819**	.840**
		Sig. (2-tailed)		.000	.000
		N	22	22	22
	**GCI**	Correlation Coefficient	.819**	1	.860**
		Sig. (2-tailed)	.000		.000
		N	22	22	22
	**CSI**	Correlation Coefficient	.840**	.860**	1
		Sig. (2-tailed)	.000	.000	
		N	22	22	22

**. Correlation is significant at the 0,01 level (2-tailed).

Source: Prepared by the authors, results obtained using SPSS.

### 4.3. Discussion

Depending on what indicators are used to measure competitiveness, the outcome will be different. The eternal question: What is the best index? Depend on the economic and political interest of countries: from the USA’s point of view, from Switzerland’s point of view or Singapore’s perspective, among other countries. These rankings are guides for relevant decisions such as the investment in countries as usually is going to be directly related to the more competitive countries instead of to the less competitive ones.

Regarding the countries, different sizes, geographical location, populations, political situations, or climate are other characteristics to take into account in order to elaborate an index. The elaboration of the competitiveness index for geographical areas by WEF in last recent years has filled a gap in the need to consider the geographical location, which in our opinion is more reasonable than producing a unique world ranking.

Moreover, in any elaboration of an index, there is subjectivity as humans are involved in the process. However, it is not questionable that inside the subjectivity, our proposed index is less manipulable for humans as no survey opinions are included.

Some results are supported by literature as in the case of Singapore. According to Loo [26], Singapore was ranked 3rd using an average between IMD-WCY and WEF-GCI during the period 2007–2011, and in the period 2009–2011 was ranking the first one. Moreover, Loo states there is still a controversial opinion concerning the different rankings provided by WEF and IMD, both Switzerland-based institutions. Nevertheless, these two indices keep being the most authoritative sources in global competitiveness. In 2012, Loo states the need for a third organization to measure competitiveness in order to conciliate both results WEF and IMD. One year later, in 2013, the first report of SolAbility-GSCI [13] only using quantitative indicators was published by this South Korean company and maintains the publication currently but from the perspective of sustainability. It could be questioned the importance in using the WEF-GCI but the transparency of the information (freely available online) and continuation in yearly published since 1979 are relevant advantages of using this index.

## 5. Conclusions

Based on a review of the methodology used by the WEF to compute the global competitiveness index, we can conclude that there is a very high percentage of qualitative data in the total data used (approximately 75%), which results in the subjectivity of the index. This subjectivity is accentuated by the arbitrary selection of weights, both for those that quantify the percentage of the different indicators in the corresponding pillar and those that indicate the importance of each pillar in the total, where the latter are also almost exclusively dependent on the country’s income level.

Additionally, the rankings provided by the WEF for the two analyzed periods include the countries for which there is no data for certain quantitative indicators (hard data) in the database the WEF provides as a basic instrument to analyze global competitiveness with the GCI. This suggests that this agency has performed some sort of data treatment to account for the missing values, either through estimation or substitution; nevertheless, the treated values are not included in said database.

This paper proposes an objective global competitiveness index that exclusively uses the data provided by the WEF and quantifies to what degree the resulting rankings are associated with those corresponding to the GCI. This index only encompasses quantitative indicators (hard data) used by the WEF and is computed by applying the multivariate exploratory technique of factor analysis, which ensures the elimination of qualitative data and, consequently, the subjectivity of the weighting.

The rankings provided by the proposed index (CSI) present a high degree of association with the rankings from the Global Competitiveness Index (GCI) for the two analyzed periods. Similarly, when limited to the European context, the association between the CSI index and the European Competitiveness Index (ECI) is not only maintained, but rather increases.

In regard to the WEF’s methodology, we lean toward a competitiveness index based on official, quantitative data that is computed using statistical and/or mathematical procedures, which considers weights that can be implicitly determined by the inherent structure of the data. We believe that this is the only way to eliminate any political biases or individual interests.

Nevertheless, this study presents some limitations such as the existence of key indicators (hard data) not considered during the analyzed period in the elaboration of WEF-GCI and therefore in the CSI; the no representation of some pillars as these pillars do not include hard data indicators.

Moreover, in any elaboration of an index, there is subjectivity as humans are involved in the process. However, as we mentioned previously it is not questionable that inside the subjectivity, our proposed index is less manipulable for humans as no survey opinions are included.

Finally, the relevant advantages of using this index are the transparency of the information of WEF-GCI (freely available online) and continuation in yearly published since 1979. It is a guide for governments, enterprises, investors, citizens among others, to manage to progress in prosperity or to achieve high living of standards.
